# Clinical Phenotypes and Comorbidity in European Sleep Apnoea Patients

**DOI:** 10.1371/journal.pone.0163439

**Published:** 2016-10-04

**Authors:** Tarja Saaresranta, Jan Hedner, Maria R. Bonsignore, Renata L. Riha, Walter T. McNicholas, Thomas Penzel, Ulla Anttalainen, John Arthur Kvamme, Martin Pretl, Pawel Sliwinski, Johan Verbraecken, Ludger Grote, ESADA Study Group

**Affiliations:** 1 Division of Medicine, Department of Pulmonary Diseases, Turku University Hospital, Turku, Finland; 2 Sleep Research Centre, Department of Physiology, University of Turku, Turku, Finland; 3 Department of Sleep Medicine, Sahlgrenska University Hospital, Gothenburg, Sweden; 4 Sahlgrenska Academy, Gothenburg University, Gothenburg, Sweden; 5 Biomedical Department of Internal and Specialistic Medicine (DIBIMIS), University of Palermo, Palermo, Italy; 6 CNR Institute of Biomedicine and Molecular Immunology, Palermo, Italy; 7 Department of Sleep Medicine, Royal Infirmary of Edinburgh, Edinburgh, United Kingdom; 8 Department of Respiratory and Sleep Medicine, St. Vincent´s University Hospital, Dublin, Ireland; 9 Conway Research Institute, University College Dublin, Dublin, Ireland; 10 Schlafmedizinisches Zentrum, Charité –Universitätsmedizin Berlin, Berlin, Germany; 11 International Clinical Research Center, St. Anne's University Hospital Brno, Brno, Czech Republic; 12 Department of ENT, Førde Central Hospital, Førde, Norway; 13 Centre for Sleep and Waking Disorders, Department of Neurology, First Faculty of Medicine, Charles University, Prague, Czech Republic; 14 Inspamed, Neurology and Sleep Laboratory, Prague, Czech Republic; 15 Institute of Tuberculosis and Lung Diseases, 4th Department of Respiratory Medicine, Warsaw, Poland; 16 Multidisciplinary Sleep Disorders Centre, Antwerp University Hospital, Antwerp, Belgium; 17 University of Antwerp, Antwerp, Belgium; Weill Cornell Medical College in Qatar, QATAR

## Abstract

**Background:**

Clinical presentation phenotypes of obstructive sleep apnoea (OSA) and their association with comorbidity as well as impact on adherence to continuous positive airway pressure (CPAP) treatment have not been established.

**Methods:**

A prospective follow-up cohort of adult patients with OSA (apnoea-hypopnoea index (AHI) of ≥5/h) from 17 European countries and Israel (n = 6,555) was divided into four clinical presentation phenotypes based on daytime symptoms labelled as excessive daytime sleepiness (“EDS”) and nocturnal sleep problems other than OSA (labelled as “insomnia”): 1) EDS (daytime+/nighttime-), 2) EDS/insomnia (daytime+/nighttime+), 3) non-EDS/non-insomnia (daytime-/nighttime-), 4) and insomnia (daytime-/nighttime+) phenotype.

**Results:**

The EDS phenotype comprised 20.7%, the non-EDS/non-insomnia type 25.8%, the EDS/insomnia type 23.7%, and the insomnia phenotype 29.8% of the entire cohort. Thus, clinical presentation phenotypes with insomnia symptoms were dominant with 53.5%, but only 5.6% had physician diagnosed insomnia. Cardiovascular comorbidity was less prevalent in the EDS and most common in the insomnia phenotype (48.9% vs. 56.8%, p<0.001) despite more severe OSA in the EDS group (AHI 35.0±25.5/h vs. 27.9±22.5/h, p<0.001, respectively). Psychiatric comorbidity was associated with insomnia like OSA phenotypes independent of age, gender and body mass index (HR 1.5 (1.188–1.905), p<0.001). The EDS phenotype tended to associate with higher CPAP usage (22.7 min/d, p = 0.069) when controlled for age, gender, BMI and sleep apnoea severity.

**Conclusions:**

Phenotypes with insomnia symptoms comprised more than half of OSA patients and were more frequently linked with comorbidity than those with EDS, despite less severe OSA. CPAP usage was slightly higher in phenotypes with EDS.

## Introduction

Obstructive sleep apnoea (OSA) is an important health problem which is associated with a reduced quality of life, as well as an increased risk of cardiovascular and metabolic co-morbidity and mortality [1–5]. The European Sleep Apnoea Database (ESADA) has recruited a large cohort of patients referred to sleep centres for evaluation of suspected OSA. This collaborative project assesses patient characteristics [6], clinical practice [7] and patient outcomes at European sleep centres.

OSA has often been considered as a uniform condition, although variable degree of daytime sleepiness, gender differences [8] in terms of OSA symptoms, or differences in the presence of comorbid insomnia [9–12], all suggesting the presence of different clinical phenotypes, were reported already more than two decades ago. More recently, systematic research has attempted to phenotype OSA based on anatomical [13] or physiological [14, 15] features or a combination of both [16]. In a previous cluster analysis the presentation of OSA varied considerably and appeared to differ in terms of symptom profile including those with insomnia-like symptoms, those with mild symptoms but a high prevalence of cardiovascular disease and the sleepy “classical” OSA type [17].

We hypothesized that distinct clinical OSA phenotypes may differ in terms of comorbidity and the adherence with nasal continuous positive airway pressure (CPAP) therapy. User friendly means to phenotype OSA are mandatory if such characterisation will be used in everyday clinical practice. Therefore, we defined the clinical presentation phenotypes based on simple-to-use and standardised tools to assess the degree of daytime as well as sleep related symptom burden in a large cohort of patients with suspected sleep apnoea.

## Methods

### Patients

Baseline and follow-up data was prospectively collected by 26 sleep laboratories in 17 European countries and Israel during the period 2007 to 2012. Data from a total of 6,555 patients (24.6% females) aged 18–80 years with an apnoea-hypopnea index (AHI) of ≥5/h were included in the final statistical analyses. Information on CPAP use in association with a first follow-up visit was available in 1,067 (16.3%) patients. Exclusion criteria were treated OSA, a limited life expectancy due to comorbidity unrelated to OSA, as well as alcohol or drug abuse within one year prior to inclusion in the study. The details of the study protocol have been published previously [6].

### Ethical considerations

The study was reviewed and specifically approved by a local ethics committee at each participating centre (Turku: Ethics Committee of the Hospital District of Southwest Finland; Gothenburg: Regionala Etikprövningsnämnden i Göteborg; Palermo: Comitato Etico Azienda Ospedaliera Universitaria Policlinico "Paolo Giaccone"; Edinburg: Lothian Research Ethics Committee; Dublin: The Ethics and Medical Research Committee of St. Vincent’s Healthcare Group; Berlin: Ethikausschuss 1 am Campus Charite Mitte, Chariteplatz 1, 10117 Berlin; Førde: Regional komite for medisinsk og helsefagleg forskningsetikk, Vest-Norge; Prague: Etická komise Všeobecné fakultní nemocnice v Praze; Warsaw: The Ethics Committee at the Institute of Tuberculosis and lung Diseases; Antwerp: Comite voor Medische Ethiek). All patients gave their written, informed consent. Patient data were coded and de-linked before entry into the central database.

### Data collection and sleep studies

Each centre adhered to the ESADA protocol and its own established clinical and diagnostic procedures with no attempt to enforce conformity on this process. Anthropometrics, medical history, medication defined by the Anatomical Therapeutic Chemical Classification (ATC) codes, daytime sleepiness, subjective as well as objective sleep data at baseline, and CPAP usage at the first follow-up visit were recorded using a structured web-based report system. Coded data were transferred and stored in a central database located at the University of Gothenburg, Sweden. In order to ensure uniform data entry procedures and data quality, a central study monitor from Gothenburg provided a training session at each centre. The study monitor has access to the complete database and has continuously monitored data quality and completeness.

The ESADA database accepted sleep studies in terms of full polysomnography (PSG) or cardio-respiratory polygraphy (PG), performed according to local practice. PSG devices had a minimum of seven, and PG devices a minimum of four channels (level 3 devices according to the American Sleep Disorders Association [18]). All sleep data were manually scored according to protocol definitions based on the rules of the American Academy of Sleep Medicine [19] before entry into the database. In PG recordings, respiratory effort related arousals (RERA) were not scored. AHI was defined as the number of apnoeas and hypopnoeas per hour of actual sleep time in PSG studies or per hour of the time period between lights off and lights on in PG studies. Subjective daytime sleepiness was assessed by the Epworth Sleepiness Scale (ESS) [20]. Subjective sleep length was reported in hours to one decimal accuracy and subjective sleep latency in minutes with one minute accuracy.

All patients were referred with a history of snoring or other symptoms suggestive of OSA. We divided patients into four categories based on subjective daytime sleepiness and nocturnal sleep complaints suggestive of insomnia: 1) excessive daytime sleepiness (EDS) without sleep complaints other than OSA (daytime+/nighttime-), 2) non-EDS, non-insomnia without sleep complaints other than OSA (daytime-/nighttime-), 3) EDS-insomnia (daytime+/nighttime+), 4) and insomnia (daytime-/nighttime+) phenotype (Table 1). EDS was defined as ESS>10. Criteria for insomnia-like symptom burden in phenotypes “EDS-insomnia” and “insomnia” were fulfilled if a patient had physician diagnosed insomnia, subjective sleep latency ≥30 min, self-reported sleep duration ≤6 h and/or use of hypnotics defined within the ATC code N05. Hence, patients receiving an “insomnia label” in this study may *not* fulfil the ICD or DSM criteria for insomnia. Still our criteria are important markers for clinically relevant insomnia.

**Table 1:** Criteria for distinct clinical phenotypes.

Criteria	Excessive daytime sleepiness ESS>10	Minimally symptomatic daytime ESS 0–10
**No sleep complaints other than OSA**	EDS phenotype (daytime+/nighttime-)	Non-EDS, non-insomnia phenotype (daytime-/nighttime-)
• **subjective sleep latency <30 min** • **self-reported sleep duration >6 h** • **no insomnia diagnosis** • **no hypnotic medication (ATC code N05)**	• N = 1,357 (20.7%)	• N = 1,690 (25.8%)
**Sleep complaints defined as patient fulfilling at least one of the following criteria:**	EDS-insomnia phenotype (daytime+/nighttime+)	Insomnia phenotype (daytime-/nighttime+)
•**insomnia diagnosis** • **subjective sleep latency ≥30 min** • **self-reported sleep duration ≤6 h** • **use of hypnotics (ATC code N05)**	• N = 1,554 (23.7%)	• N = 1,954 (29.8%)

ATC = Anatomical Therapeutic Chemical Classification, EDS = excessive daytime sleepiness, ESS = Epworth Sleepiness Scale, OSA = obstructive sleep apnoea

### Statistics

The data were analysed both as an entire file and as a split file according to sleep study method (PSG or PG). Data are presented as mean ± standard deviation or as frequency (%). Comparisons among the groups were performed using independent samples t-tests and ANOVA, or Wilcoxon Two-Sample Test or Kruskall-Wallis test as appropriate for continuous variables, or the Chi-square tests for categorical variables. Impact of age and gender on different prevalence of cardiovascular and psychiatric diseases among clinical presentation phenotypes was analysed by logistic regression. Impact of age, BMI, gender, AHI, and phenotypes on CPAP usage was analysed using linear regression. Statistical analyses were performed using IBM SPSS Statistics 22.0 (Armonk, NY, USA: IBM Corp.). Adjustments for multiple comparisons were not performed but p-value <0.01 was considered statistically significant. All tests were two-sided.

## Results

### Distribution of OSA phenotypes

The EDS phenotype comprised 1,357 patients (20.7%), the EDS-insomnia phenotype 1,554 patients (23.7%), the non-EDS, non-insomnia phenotype 1,690 patients (25.8%), and the insomnia phenotype 1,954 patients (29.8%) of the entire cohort (Table 1). Clinical presentation phenotypes with sleep complaints suggestive of insomnia (EDS-insomnia type and insomnia phenotype) comprised 53.5% and those with significant daytime hypersomnolence (EDS and EDS-insomnia type) 44.4% of the entire cohort. Physician diagnosed insomnia was rare (4.9% of patients in the EDS-insomnia group and 6.1% in the insomnia group, Table 1). A use of ATC code N05 medication was reported in 11.8% and 14.6% of patients with EDS-insomnia or insomnia phenotype. The number of patients with physician diagnosed insomnia and/or using hypnotics was 611 (17.6% of total number of patients labeled as insomnia or EDS-insomnia phenotype). Prolonged sleep latency (≥30 min) was reported in 56.3% and 63.4%, and short sleep duration (≤6hr/night) in 62.4% and 57.2%, of patients with sleep related symptoms (EDS-insomnia or insomnia phenotype, respectively). The number of patients included in the insomnia phenotypes purely based on the criterion of sleep duration ≤6 h was 1067 (30.4%). In patients with EDS (combined EDS and EDS-insomnia phenotypes), short sleep duration (≤6hr/night) was prevalent in 33.3% of patients. Self-reported and objectively measured sleep duration behaved similarly. In the PSG cohort, both self-reported sleep duration (6 hr 16 min vs. 7 hr 42 min, p<0.001) and PSG-based total sleep time (6 hr 11 min vs. 6 hr 33 min, p<0.001) were shorter within the two insomnia phenotypes compared with the patients within the two hypersomnia phenotypes. In the entire PSG group, self-reported average sleep duration was 7 hr 0 min and TST 6 hr 20 min (p<0.001). In those labeled as EDS-insomnia or insomnia phenotypes, self-reported sleep duration was 6 hr 16 min and PSG-based total sleep time (6 hr 11 min, p = 0.070).

### Anthropometric and clinical data

Insomnia phenotype patients were older than EDS phenotype patients (54.6 vs 51.5 years, p<0.001, Table 2). The two insomnia phenotypes were more likely to be female, whereas the non-EDS, non-insomnia phenotype had the highest male proportion (Table 2). Furthermore, the two EDS phenotypes had more severe sleep apnoea than the insomnia phenotype (AHI 35.0±25.5/h and 33.2±25.5/h vs. 27.9±22.5/h, p<0.001, respectively). There was only a minor difference in BMI between the EDS-insomnia type and the non-EDS, non-insomnia phenotype (32.7 kg/m^2^ vs. 31.1 kg/m^2^, p = 0.003, Table 2). Systolic and diastolic blood pressure, alcohol consumption or the relative proportion of current smoking did not differ between the clinical presentation phenotypes (Table 2). Characteristics of the OSA phenotypes did not differ in a clinically relevant manner between the two sub-cohorts defined according to the sleep study method (PSG or PG) (S1 Table and S2 Table).

**Table 2:** Clinical characteristics of the four different OSA clinical phenotypes. Data presented as an average (95% CI).

	EDS	EDS-insomnia	Non-EDS, non-insomnia	Insomnia					
					P-value	P-value	P-value	P-value	P-value
					Across all	Insomnia vs. EDS	Insomnia vs. EDS-insomnia	EDS-insomnia vs. EDS	EDS vs. non-EDS, non-insomnia
				
**Female gender (%)**	22.8 (20.6–25.0)	28.2 (26.0–30.4)	17.0 (15.2–18.8)	29.6 (27.6–31.6)	**<0.001**	**<0.001**	0.389	**0.001**	**<0.001**
**Age (years)**	51.5 (50.8–52.1)	52.2 (51.7–52.8)	52.8 (52.2–53.4)	54.6 (54.1–55.2)	**<0.001**	**<0.001**	**<0.001**	0.067	**0.002**
**BMI (kg/m**^**2**^**)**	31.9 (31.6–32.2)	32.7 (32.3–33.0)	31.2 (30.9–31.5)	31.3 (31.0–31.6)	**<0.001**	**0.009**	**<0.001**	**0.001**	**0.003**
**BMI > 30 kg/m**^**2**^ **(%)**	56.3 (53.7–58.9)	61.4 (59.0–63.8)	51.5 (49.1–53.9)	52.3 (50.1–54.5)	**<0.001**	0.023	**<0.001**	**0.006**	**0.008**
**Waist (cm)**	108.8 (108.0–109.6)	110.5 (109.7–111.3)	108.1 (107.4–108.8)	108.0 (107.3–108.7)	**<0.001**	0.151	**<0.001**	**0.003**	0.225
**Neck (cm)**	42.0 (41.7–42.2)	42.1 (41.9–42.4)	42.1 (41.9–42.3)	41.5 (41.3–41.7)	**<0.001**	**0.002**	**<0.001**	0.33	0.326
**Systolic BP (mmHg)**	134.4 (133.5–135.3)	135.4 (134.5–136.3)	134.8 (134.0–135.6)	134.9 (134.1–135.7)	0.519	0.422	0.431	0.137	0.52
**Diastolic BP (mmHg)**	83.0 (82.5–83.6)	83.0 (82.4–83.5)	82.7 (82.1–83.2)	83.3 (82.7–83.8)	0.475	0.585	0.43	0.827	0.361
**Current smokers (%)**	24.2 (21.9–26.5)	25.8 (23.6–28.0)	22.0 (20.0–24.0)	24.6 (22.7–26.5)	0.072	0.805	0.389	0.303	0.152
**Alcohol consumption (units/week)**	4.5 (4.1–5.0)	4.4 (4.0–4.9)	4.7 (4.3–5.1)	4.1 (3.7–4.5)	0.238	0.176	0.296	0.803	0.566
** **					** **	** **	** **		
**AHI #/h** PSG n = 3216	37.4 (35.4–39.3)	35.5 (33.6–37.4)	34.4 (32.9–36.0)	32.0 (30.5–33.5)	**<0.001**	**<0.001**	**0.004**	0.188	0.021
PG n = 3339	32.7 (30.8–34.6)	31.3 (29.6–32.9)	27.1 (25.7–28.6)	24.0 (22.8–25.3)	**<0.001**	**<0.001**	**<0.001**	0.258	**<0.001**
**ODI4 #/h** PSG n = 3101	26.6 (24.6–28.5)	27.2 (25.3–29.2)	23.7 (22.1–25.4)	23.6 (22.0–25.1)	**0.004**	0.018	**0.004**	0.645	0.028
PG n = 2885	28.3 (26.3–30.2)	27.2 (25.5–29.0)	22.0 (20.5–23.5)	19.7 (18.5–21.0)	**<0.001**	**<0.001**	**<0.001**	0.446	**<0.001**
**Average subjective sleep latency (min)**	9.1 (8.8–9.4)	32.2 (30.7–33.8)	10.2 (9.9–10.4)	37.4 (35.8–39.0)	**<0.001**	**<0.001**	**<0.001**	**<0.001**	**<0.001**
**Average subjective sleep length (hr)**	7.7 (7.7–7.8)	6.2 (6.1–6.2)	7.6 (7.6–7.7)	6.4 (6.3–6.5)	**<0.001**	**<0.001**	**<0.001**	**<0.001**	**0.003**
**Intake of hypnotics (ATC N05 or N06) (%)**	6.2 (4.9–7.5)	21.0 (19.0–23.0)	4.0 (3.1–4.9)	21.2 (19.4–23)	**<0.001**	**<0.001**	0.868	**<0.001**	**0.005**
**Subjects diagnosed with insomnia (%)**	0 (0–0)	4.9 (3.8–6.0)	0 (0–0)	6.1 (5.0–7.2)	**<0.001**	**<0.001**	0.135	**<0.001**	1.000
**ESS score**	14.9 (14.7–15.0)	14.8 (14.7–15.0)	6.1 (6.0–6.2)	6.0 (5.9–6.1)	**<0.001**	**<0.001**	**<0.001**	0.756	**<0.001**

AHI = apnoea-hypopnoea index, ATC = Anatomical Therapeutic Chemical Classification, BMI = body mass index, BP = blood pressure, EDS = excessive daytime sleepiness, ESS = Epworth Sleepiness Scale, ODI4 = oxygen desaturation index (drops of arterial oxyhemoglobin saturation 4% or more per hour), PG = cardio-respiratory polygraphy, PSG = polysomnography

### Comorbidities

The analysis identified several differences across the comorbidity spectrum in the four OSA phenotypes (Table 3).

**Table 3:** Prevalence of comorbidities and the use of CPAP at the first follow-up visit in the four different OSA phenotypes. Data presented as an average (95% CI).

	EDS	EDS-insomnia	Non-EDS, non-insomnia	Insomnia	P-value	P-value	P-value	P-value	P-value	P-value
					Across all	Insomnia vs. EDS	Insomnia vs. EDS-insomnia	EDS-insomnia vs. EDS	EDS vs. non-EDS, non-insomnia	Insomnia vs. non-EDS, non-insomnia
					
**Cardiovascular disease (%)**	48.9 (46.2–51.6)	53.0 (50.5–55.5)	52.0 (49.6–54.4)	56.8 (54.6–59.0)	**<0.001**	**<0.001**	0.026	0.026	0.087	**0.004**
**Metabolic disease (%)**	36.5 (33.9–39.1)	38.0 (35.6–40.4)	38.6 (36.3–40.9)	39.2 (37.0–41.4)	0.445	0.118	0.464	0.42	0.244	0.708
**Pulmonary disease (%)**	13.7 (11.9–15.5)	15.8 (14.0–17.6)	12.2 (10.6–13.8)	16.0 (14.4–17.6)	**0.005**	0.075	0.889	0.129	0.253	**0.001**
**Psychiatric disease (%)**	8.7 (7.2–10.2)	14.5 (12.7–16.3)	5.0 (4.0–6.0)	12.6 (11.1–14.1)	**<0.001**	**<0.001**	0.1	**<0.001**	**<0.001**	**<0.001**
**CPAP usage at the 1st control visit (h/d)**	4.8 (4.5–5.0)	4.4 (4.1–4.7)	4.7 (4.4–5.0)	4.4 (4.1–4.7)	0.126	0.069	0.990	0.055	0.737	0.149

EDS = excessive daytime sleepiness, CPAP = continuous positive airway pressure

Cardiovascular comorbidity was less prevalent in the EDS patients and most common in the insomnia phenotype (48.9% vs. 56.8%, p<0.001) in univariate analysis. When controlling for age, gender and BMI, the insomnia phenotype had higher cardiovascular comorbidity (logistic regression, p = 0.022) (Table 4). The highest prevalence of psychiatric comorbidity was found in the EDS-insomnia (14.5%) and insomnia (12.6%) groups (Table 3). These phenotypes independently and significantly explained the difference in psychiatric comorbidity, when controlling for age, gender and BMI (Table 5). Insomnia phenotype patients also tended to have a higher prevalence of pulmonary comorbidities despite less severe OSA, when compared with the EDS phenotype (Table 3). When compared with the non-EDS, non-insomnia group, cardiovascular, pulmonary and psychiatric diseases were more prevalent in the insomnia group (Table 3).

**Table 4:** Predictors of higher cardiovascular comorbidity compared to the prevalence of cardiovascular diseases in the EDS-phenotype: Independent influence of the phenotype, age, gender and BMI.

Factor	HR	Confidence interval	P-value
**Constant**	0.001		**<0.001**
**EDS phenotype**	1.000		0.140
**EDS-insomnia phenotype**	1.086	0.923–1.278	0.321
**Non-EDS, non-insomnia phenotype**	1.082	0.922–1.271	0.335
**Insomnia phenotype**	1.201	1.027–1.404	**0.022**
**Age**	1.089	1.083–1.095	**<0.001**
**Gender (male)**	1.348	1.186–1.533	**<0.001**
**BMI**	1.090	1.080–1.100	**<0.001**

BMI = body mass index, EDS = excessive daytime sleepiness

**Table 5:** Predictors of higher psychiatric comorbidity compared to the prevalence of psychiatric diseases in the EDS-phenotype: Independent influence of the phenotype, age, gender and BMI.

Factor	HR	Confidence interval	P-value
**Constant**	0.205		**<0.001**
**EDS phenotype**	1.000		**<0.001**
**EDS-insomnia phenotype**	1.715	1.350–2.179	**<0.001**
**Non-EDS, non-insomnia phenotype**	0.611	0.457–0.818	**0.001**
**Insomnia phenotype**	1.504	1.188–1.905	**0.001**
**Age**	0.986	0.979–0.993	**<0.001**
**Gender (male)**	0.389	0.328–0.461	**<0.001**
**BMI**	1.019	1.007–1.031	**0.001**

BMI = body mass index, EDS = excessive daytime sleepiness

Within the two insomnia phenotypes those patients with short sleep duration (≤6 h) had a higher prevalence of psychiatric comorbidity compared with the normal/long sleepers (>6 h) (18.7% vs. 9.1%, p<0.001), whereas the prevalence of cardiovascular (55.8% vs. 54.8%), pulmonary (16.5% vs. 15.0%), or metabolic disease (38.6% vs. 38.8%) did not differ between the two groups.

### CPAP compliance

Data on CPAP adherence at the first follow-up visit were available in 1,067 patients. CPAP usage tended to be lower among phenotypes linked to insomnia although the differences did not reach statistical significance (p = 0.055 and 0.069, Table 3). In a linear regression analysis, the EDS phenotype tended to associate (p = 0.053) with higher CPAP use (24.2 min/d) when controlling for age, BMI and gender in the model. This tendency towards higher CPAP usage in the EDS phenotype remained (22.7 min/d, p = 0.069) (Table 6) when ODI4 was added to the model.

**Table 6:** Predictors of CPAP compliance at the first follow up visit: Independent influence of the four clinical phenotypes. OSA severity defined as oxygen desaturation index included in the model.

Factor	HR	Confidence interval	P-value
**Constant**	2.459	1.207–3.710	**<0.001**
**Age**	0.015	0.002–0.029	0.023
**BMI**	0.026	0.000–0.052	0.047
**Gender**	0.072	-0.282–0.426	0.690
**ODI4**	0.007	0.000–0.015	0.044
**Phenotype**			
• **EDS** • **EDS-insomnia** • **Non-EDS, non-insomnia** • **Insomnia**	0.379	-0.029–0.787	0.069
	0.000	NA	NA
	0.324	-0.094–0.741	0.129
	0.037	-0.366–0.440	0.857

ODI4 = oxygen desaturation index (drops of arterial oxyhemoglobin saturation 4% or more per hour), BMI = body mass index, EDS = excessive daytime sleepiness

## Discussion

The analysis by clinical phenotype classification in this large European sleep apnoea cohort has provided three major findings. First, insomnia, as defined in the study, is a common phenotypic characteristic that exceeds daytime hypersomnia in terms of prevalence. Secondly, the prevalence of comorbidities was high and symptom-based OSA phenotypic subgroups differed in terms of reported comorbidity. Cardiovascular, pulmonary and psychiatric comorbidities were more prevalent among the OSA phenotypes with comorbid insomnia symptoms. Thirdly, our data suggest that adherence to CPAP therapy might be influenced or even predicted by clinical presentation phenotype. However, prospective outcome studies are needed to more precisely assess the value of defining clinical presentation phenotype with respect to OSA management.

### The general concept of phenotyping

Although gender-specific differences in symptoms [8] and differences in the presence of comorbid insomnia [9, 10] are recognised in OSA, the importance of phenotyping OSA patients, particularly in the context of OSA treatment, has only lately been acknowledged [13, 14]. In fact, steps to formalise the phenotyping process have been taken [17, 21], but a consensus on detailed principles for classification remains to be established. The ESADA cohort provides an excellent opportunity to probe some proposed phenotypic characteristics although the database is not detailed enough to permit phenotyping according to functional (neurocognitive, respiratory control) or structural characteristics. The clinical characteristics (ESS score, subjective sleep duration and sleep latency, physician-diagnosed sleep disorder, use of hypnotics) used for phenotyping patients in the present study were those considered to be readily available to clinicians treating patients with OSA.

### High prevalence of insomnia related phenotypes

The frequency of reported insomnia symptoms in different OSA cohorts varies between 39% and 55% and occult OSA has been reported in 29% to 67% of the cases with diagnosis of clinical insomnia [22]. In line with those studies, our current data show that shortened sleep time and increased sleep latency each were reported by approximately one third of patients with verified OSA. Not less than 56% of the cohort were labelled as an EDS-insomnia or insomnia phenotype. However, the conventional description of a typical OSA patient has focused on symptoms of increased daytime sleepiness [23, 24]. Interestingly, one third of the OSA patients with hypersomnia had a short sleep time, which may aggravate the burden of OSA with respect to cognitive function and vigilance. Only a minority of patients with shortened sleep and increased sleep latency were diagnosed and/or treated for insomnia symptoms. Our data strongly suggest that the conventional image of a sleepy OSA patient may not represent the dominant phenotype in clinical practice at European sleep centres. A more differentiated classification of phenotypes based on patient complaints may definitively be needed in the clinical management of OSA patients.

### Association between phenotypes and comorbidities

The prevalence of comorbidities including cardiovascular disease [3, 25, 26], metabolic syndrome [27], type 2 diabetes mellitus [4, 5, 28], mood disorders [29] or obstructive lung diseases [30] was, as expected, high in the ESADA cohort. An interesting finding was the distribution of comorbidities across the OSA phenotypes. Patients with significant hypersomnia (EDS and EDS-insomnia phenotypes) were slightly younger and had more severe OSA compared to those with mild or no daytime sleepiness (non-EDS, non-insomnia and insomnia phenotypes). However, patients with the EDS phenotype had the lowest prevalence of cardiovascular disease among the clinical presentation phenotypes. Conversely, patients reporting difficulty in initiating and/or maintaining sleep (EDS-insomnia or insomnia phenotype) more frequently had cardiovascular, pulmonary and/or psychiatric comorbidities. These results are in line with the findings in an Icelandic cohort of OSA patients phenotyped according to quite similar criteria [17]. Moreover, in a Swedish population-based random sample of women with OSA, daytime sleepiness and hypertension appeared to represent two different phenotypes [31]. A recent cluster analysis using the data from the French National registry of Sleep Apnoea identified six clusters [32]. A high prevalence of comorbidities was found in the cluster of the obese elderly minimally symptomatic in line with our findings. However, contrary to our results, they also reported a high prevalence of comorbidity among the obese middle-aged symptomatic patients.

The exact reason for the differences in the comorbidity spectra remains unexplained in our study. However, differences may be a result of referral bias. Potential explanations include an increased time lag from disease start to final diagnosis and treatment in “asymptomatic” OSA, causing more exposure to harmful cardiovascular consequences of OSA in this group [17]. Older age in the insomnia phenotype may at least in part explain the differences. Insomnia symptoms may be a consequence and not necessarily the cause of comorbidities. However, our cross-sectional baseline data does not allow us to explore the potential causality of this association. Further, in our database, we do not have data on how well e.g. pulmonary diseases or heart failure were controlled in a medical sense. There may also be functional mechanisms explaining the increased cardiovascular comorbidity associated with insomnia symptoms. For instance, the insomnia and EDS-insomnia phenotype may have elevated adrenergically mediated alertness manifested as long sleep latency or short self-reported sleep duration. This hypothesis is supported by observations among OSA and insomnia patients. Higher sympathetic activity has been observed in non-sleepy patients with severe OSA compared to those with excessive daytime sleepiness [33] as well as in primary insomniacs compared with good sleepers [34]. In fact, some previous studies have linked cardiovascular comorbidity to non-sleepy OSA like in patients with peripheral arterial disease [35], in perimenopausal women [36] or in depression [37].

### Treatment outcome: CPAP adherence

Adequate adherence to CPAP treatment strongly influences clinical outcome. Symptoms of insomnia in OSA patients have been reported to be associated with lower CPAP adherence [12, 38, 39]. This is in line with our findings showing slightly lower adherence at the first follow-up visit among clinical presentation phenotypes including insomnia symptoms. When taking age, gender, BMI, and OSA severity into account, the association of adherence with the different phenotypes remained unchanged. In this highly selected subsample of CPAP users, the relatively good short-term CPAP adherence is likely to be predictive of good long-term adherence [40].

### Strengths and limitations

Despite inherent limitations in the data report format, the ESADA cohort provides a unique opportunity to document the practise of assessing and treating OSA in different areas of Europe. Female patients are well represented in the cohort allowing also consideration of gender-related issues. The centralised data monitoring and web-based report format provide conformity in the reported data sets. A specific methodological weakness resides in the locally used diagnostic routines applied at participating centres, which may lead to differences in the reported sleep related variables and comorbidities. A major limitation in our study is the broad definition of insomnia, which does not fulfil the ICD or DSM criteria and therefore may lead to overestimation of the prevalence of “true” insomnia. However, the finding that even symptoms of insomnia in OSA patients are associated with increased comorbidity would still be relevant in this context. Moreover, our findings do not suggest a bias related to the type of sleep study used, since the phenotypes were equally represented in both the PG and PSG cohorts. The number of patients with follow-up data is limited. This reflects the economic constraints in many centres which prevents regular follow-up of patients after the initiation of CPAP therapy. Unfortunately our resources did not allow us to collect the follow-up data outside the sleep centres e.g. from health care providers. Finally, although the database does not allow for comprehensive analysis of the effects of sociodemographic factors on referral patterns or patient outcomes, it represents by far the most comprehensive description of clinical characteristics among European OSA patients.

### Clinical implications and future research

Our findings emphasise that OSA can occur in a wide range of “non-traditional” presentations. This observation serves to alert primary health care providers to the possibility of OSA also in subgroups of slim patients, non-sleepy short-sleepers, and women.

Improved classification of phenotypes is warranted in order to better distinguish between insomnia, hypersomnia and non-EDS, non-insomnia OSA phenotypes. Identification of such differences in phenotypes might lead to an improvement in clinical practice by recognising and tailoring treatment to the specific phenotype. In addition, treatment effects on outcomes like blood pressure, traffic accident rate or mood disturbance are likely to differ considerably depending on OSA phenotype. Indeed, small studies have already demonstrated that cognitive behavioural treatment of comorbid insomnia increased CPAP compliance and improved morning restfulness and daytime alertness in patients with OSA [11, 12].

## Conclusions

Insomnia-like symptoms including difficulty initiating or maintaining sleep dominated clinical presentation of patients with OSA in this large pan-European cohort. Daytime sleepiness was a less frequent presenting complaint. In addition, clinical presentation phenotypes defined according to sleepiness and insomnia-like symptoms were associated with considerable differences in comorbidity. A high prevalence of particularly cardiovascular, pulmonary and psychiatric comorbidity among two insomnia phenotypes with and without EDS should alert clinicians to identify this type of OSA patients. CPAP adherence tended to differ among phenotypes and prospective studies of patients treated with various modalities of CPAP or mandibular advancement devices may benefit from stratification according to phenotype to increase our understanding of which therapy is most likely to be of greatest benefit.

## Supporting Information

S1 Table Clinical characteristics of the four different OSA clinical phenotypes. (DOCX)

S2 Table Sleep and sleepiness characteristics of the four different clinical phenotypes. (DOCX)
