# Supplementary material for: Clinical Phenotypes and Comorbidity in European Sleep Apnoea Patients
Source: PLoS One. 2016 Oct 4;11(10):e0163439. doi: 10.1371/journal.pone.0163439 (PMC5049787; doi:10.1371/journal.pone.0163439)
Supplement: S1 Table — (DOCX) [file pone.0163439.s001.docx]

**S1 Table.** Clinical characteristics of the four different OSA clinical phenotypes.

|  | **Cohort** | **Overall** | **EDS** | **EDS-insomnia** | **Non-EDS, non-insomnia** | **Insomnia** | **P-value** | **P-value** | **P-value** | **P-value** |
| --- | --- | --- | --- | --- | --- | --- | --- | --- | --- | --- |
|  |  |  |  |  |  |  | **Across all** | **Insomnia vs. EDS** | **Insomnia vs. EDS-insomnia** | **EDS-insomnia vs. EDS** |
|  |  | **PSG N = 3216** | **PSG N = 672** | **PSG N = 711** | **PSG N = 887** | **PSG N = 946** |  |  |  |  |
|  |  | **PG N = 3339** | **PG N = 685** | **PG N = 843** | **PG N = 803** | **PG N = 1008** |  |  |  |  |
|  |  |  |  |  |  |  |  |  |  |  |
| **Females (%)** | PSG | 23.7 (22.2-25.2) | 23.1 (19.9-26.3) | 25.2 (22.0-28.4) | 17.1 (14.6-19.6) | 29.3 (26.4-32.2) | **<0.001** | **0.006** | 0.067 | 0.379 |
|  | n = 3216 |  |  |  |  |  |  |  |  |  |
|  | PG | 25.5 (24.0-27.0) | 22.6 (19.5-25.7) | 30.8 (27.7-33.9) | 16.8 (14.2-19.4) | 30.0 (27.2-32.8) | **<0.001** | **0.001** | 0.685 | **<0.001** |
|  | n = 3339 |  |  |  |  |  |  |  |  |  |
| **Age (years)** | PSG | 52.4 (52.0-52.8) | 50.7 (49.8-51.6) | 51.8 (50.9-52.6) | 52.1 (51.3-52.9) | 54.3 (53.5-55) | **<0.001** | **<0.001** | **<0.001** | 0.092 |
|  | n = 3216 |  |  |  |  |  |  |  |  |  |
|  | PG | 53.5 (53.1-53.9) | 52.2 (51.3-53.1) | 52.6 (51.9-53.4) | 53.6 (52.7-54.5) | 55.0 (54.2-55.7) | **<0.001** | **<0.001** | **<0.001** | 0.441 |
|  | n = 3339 |  |  |  |  |  |  |  |  |  |
| **BMI (kg/m^2^)** | PSG | 31.3 (31.1-31.6) | 31.5 (31.0-31.9) | 31.9 (31.4-32.5) | 30.8 (30.5-31.2) | 31.2 (30.8-31.6) | **0.006** | 0.423 | 0.03 | 0.184 |
|  | n = 3213 |  |  |  |  |  |  |  |  |  |
|  | PG | 32.1 (31.9-32.3) | 32.3 (31.8-32.8) | 33.3 (32.9-33.8) | 31.6 (31.2-32.1) | 31.4 (31.0-31.8) | **<0.001** | **0.004** | **<0.001** | **0.004** |
|  | n = 3335 |  |  |  |  |  |  |  |  |  |
|  | PSG | 52.6 (50.9-54.3) | 53.1 (49.3-56.9) | 56.8 (53.2-60.4) | 49.3 (46.0-52.6) | 52.2 (49.0-55.4) | 0.029 | 0.762 | 0.065 | 0.176 |
| **BMI > 30 kg/m^2^ (%)** | n = 3213 |  |  |  |  |  |  |  |  |  |
|  | PG | 57.4 (55.7-59.1) | 59.4 (55.7-63.1) | 65.3 (62.1-68.5) | 53.9 (50.4-57.4) | 52.3 (49.2-55.4) | **<0.001** | **0.005** | **<0.001** | 0.019 |
|  | n = 3335 |  |  |  |  |  |  |  |  |  |
| **BMI > 35 kg/m^2^ (%)** | PSG | 22.8 (21.3-24.3) | 21.3 (18.2-24.4) | 25.8 (22.6-29.0) | 20.0 (17.4-22.6) | 24.3 (21.6-27.0) | 0.021 | 0.168 | 0.528 | 0.057 |
|  | n = 3213 |  |  |  |  |  |  |  |  |  |
|  | PG | 27.9 (26.4-29.4) | 29.2 (25.8-32.6) | 37.2 (33.9-40.5) | 22.3 (19.4-25.2) | 23.7 (21.1-26.3) | **<0.001** | 0.013 | **<0.001** | **0.001** |
|  | n = 3335 |  |  |  |  |  |  |  |  |  |
| **Neck (cm)** | PSG | 41.6 (41.5-41.8) | 41.7 (41.4-42.1) | 41.6 (41.3-42.0) | 41.9 (41.6-42.2) | 41.3 (41.0-41.6) | 0.022 | 0.035 | 0.104 | 0.658 |
|  | n = 3213 |  |  |  |  |  |  |  |  |  |
|  | PG | 42.1 (42.0-42.3) | 42.2 (41.8-42.5) | 42.5 (42.2-42.9) | 42.3 (42.1-42.6) | 41.6 (41.4-41.9) | **<0.001** | 0.016 | **<0.001** | 0.144 |
|  | n = 3335 |  |  |  |  |  |  |  |  |  |
| **Waist (cm)** | PSG | 107.4 (106.9-108.0) | 107.1 (106.0-108.1) | 108.7 (107.5-109.9) | 106.9 (105.9-107.8) | 107.3 (106.3-108.3) | 0.075 | 0.75 | 0.075 | 0.042 |
|  | n = 3167 |  |  |  |  |  |  |  |  |  |
|  | PG | 110.0 (109.5-110.6) | 110.4 (109.2-111.6) | 112.0 (110.9-113.1) | 109.5 (108.4-110.5) | 108.6 (107.7-109.5) | **<0.001** | 0.018 | **<0.001** | 0.052 |
|  | n = 3339 |  |  |  |  |  |  |  |  |  |
| **Waist-hip ratio** | PSG | 0.98 (0.98-0.98) | 0.97 (0.97-0.98) | 0.99 (0.98-0.99) | 0.98 (0.97-0.98) | 0.98 (0.97-0.98) | 0.032 | 0.614 | 0.026 | 0.01 |
|  | n = 3167 |  |  |  |  |  |  |  |  |  |
|  | PG | 0.98 (0.98-0.98) | 0.98 (0.98-0.99) | 0.98 (0.98-0.99) | 0.98 (0.98-0.99) | 0.97 (0.97-0.98) | 0.025 | 0.019 | 0.062 | 0.623 |
|  | n = 3339 |  |  |  |  |  |  |  |  |  |
| **Alcohol (units/week)** | PSG | 3.6 (3.4-3.9) | 3.6 (3.1-4.1) | 3.6 (3.0-4.1) | 3.9 (3.4-4.3) | 3.5 (3.0-4.0) | 0.719 | 0.842 | 0.829 | 0.983 |
|  | n = 2953 |  |  |  |  |  |  |  |  |  |
|  | PG | 5.3 (5.0-5.7) | 5.5 (4.8-6.2) | 5.2 (4.5-6.0) | 5.7 (5.1-6.4) | 4.9 (4.2-5.5) | 0.305 | 0.169 | 0.452 | 0.592 |
|  | N=2616 |  |  |  |  |  |  |  |  |  |
| **Systolic BP (mmHg)** | PSG | 131.4 (130.8-131.9) | 131.2 (129.9-132.4) | 132 (130.8-133.3) | 130.7 (129.7-131.7) | 131.7 (130.6-132.8) | 0.381 | 0.548 | 0.67 | 0.335 |
|  | n = 3216 |  |  |  |  |  |  |  |  |  |
|  | PG | 138.3 (137.7-138.9) | 137.6 (136.3-138.9) | 138.2 (137.0-139.4) | 139.4 (138.1-140.6) | 137.9 (136.8-139.0) | 0.227 | 0.691 | 0.749 | 0.502 |
|  | n = 3339 |  |  |  |  |  |  |  |  |  |
| **Diastolic BP (mmHg)** | PSG | 81.2 (80.9-81.6) | 81.2 (80.4-82.0) | 80.9 (80.1-81.8) | 81.0 (80.3-81.7) | 81.7 (81.0-82.5) | 0.399 | 0.305 | 0.159 | 0.702 |
|  | n = 3216 |  |  |  |  |  |  |  |  |  |
|  | PG | 84.7 (84.3-85.1) | 84.9 (84.1-85.7) | 84.7 (83.9-85.4) | 84.5 (83.7-85.4) | 84.7 (84.0-85.4) | 0.944 | 0.719 | 0.947 | 0.682 |
|  | n = 3339 |  |  |  |  |  |  |  |  |  |
