# Supplementary material for: Clinical Phenotypes and Comorbidity in European Sleep Apnoea Patients
Source: PLoS One. 2016 Oct 4;11(10):e0163439. doi: 10.1371/journal.pone.0163439 (PMC5049787; doi:10.1371/journal.pone.0163439)
Supplement: S2 Table — (DOCX) [file pone.0163439.s002.docx]

**S2 Table.** Sleep and sleepiness characteristics of the four different clinical phenotypes.

|  | **Cohort** | **Overall** | **EDS** | **EDS-insomnia** | **Non-EDS, non-insomnia** | **Insomnia** | **P-value** | **P-value** | **P-value** | **P-value** |
| --- | --- | --- | --- | --- | --- | --- | --- | --- | --- | --- |
|  |  |  |  |  |  |  | **Across all** | **Insomnia vs. EDS** | **Insomnia vs. EDS-insomnia** | **EDS-insomnia vs. EDS** |
|  |  | **PSG N = 3216** | **PSG N = 672** | **PSG N = 711** | **PSG N = 887** | **PSG N = 946** |  |  |  |  |
|  |  | **PG N = 3339** | **PG N = 685** | **PG N = 843** | **PG N = 803** | **PG N = 1008** |  |  |  |  |
|  |  |  |  |  |  |  |  |  |  |  |
| **ESS** | PSG | 9.8 (9.6-10.0) | 14.8 (14.6-15.1) | 14.7 (14.5-14.9) | 6.1 (5.9-6.3) | 5.9 (5.7-6.1) | **<0.001** | **<0.001** | **<0.001** | 0.506 |
|  | n = 3216 |  |  |  |  |  |  |  |  |  |
|  | PG | 10.1 (10.0-10.3) | 14.9 (14.7-15.1) | 14.9 (14.7-15.1) | 6.1 (5.9-6.3) | 6.1 (6.0-6.3) | **<0.001** | **<0.001** | **<0.001** | 0.878 |
|  | n = 3339 |  |  |  |  |  |  |  |  |  |
| **ESS > 10 (%)** | PSG | 43.0 (41.3-44.7) | 100 (100-100) | 100 (100-100) | 0 (0-0) | 0 (0-0) | **<0.001** | **<0.001** | **<0.001** | 1.000 |
|  | n = 3216 |  |  |  |  |  |  |  |  |  |
|  | PG | 45.8 (44.1-47.5) | 100 (100-100) | 100 (100-100) | 0 (0-0) | 0 (0-0) | **<0.001** | **<0.001** | **<0.001** | 1.000 |
|  | n = 3339 |  |  |  |  |  |  |  |  |  |
| **Average subjective sleep length (h)** | PSG | 7.0 (6.9-7.0) | 7.7 (7.7-7.8) | 6.2 (6.1-6.3) | 7.7 (7.6-7.7) | 6.3 (6.2-6.4) | **<0.001** | **<0.001** | 0.057 | **<0.001** |
|  | n = 3166 |  |  |  |  |  |  |  |  |  |
|  | PG | 6.9 (6.9-7.0) | 7.7 (7.7-7.8) | 6.1 (6.0-6.3) | 7.6 (7.5-7.7) | 6.4 (6.3-6.5) | **<0.001** | **<0.001** | **<0.001** | **<0.001** |
|  | n = 3272 |  |  |  |  |  |  |  |  |  |
| **Average subjective sleep length ≤ 6 h (%)** | PSG | 31.6 (30.0-33.2) | 0 (0-0) | 64.4 (60.8-68.0) | 0 (0-0) | 60.6 (57.4-63.8) | **<0.001** | **<0.001** | 0.119 | **<0.001** |
|  | n = 3166 |  |  |  |  |  |  |  |  |  |
|  | PG | 31.1 (29.5-32.7) | 0 (0-0) | 60.6 (57.2-64.0) | 0 (0-0) | 54.0 (50.9-57.1) | **<0.001** | **<0.001** | **0.005** | **<0.001** |
|  | n = 3272 |  |  |  |  |  |  |  |  |  |
| **Average subjective sleep latency (min)** | PSG | 21.8 (20.9-22.7) | 9.7 (9.3-10.2) | 31.1 (28.8-33.4) | 10.7 (10.4-11.1) | 34.8 (32.5-37) | **<0.001** | **<0.001** | 0.028 | **<0.001** |
|  | n = 3128 |  |  |  |  |  |  |  |  |  |
|  | PG | 24.1 (23.1-25.1) | 8.5 (8.0-8.9) | 33.2 (31.1-35.4) | 9.5 (9.2-9.9) | 39.9 (37.7-42.1) | **<0.001** | **<0.001** | **<0.001** | **<0.001** |
|  | n = 3247 |  |  |  |  |  |  |  |  |  |
| **Average subjective sleep latency ≥ 30 min (%)** | PSG | 29.2 (27.6-30.8) | 0 (0-0) | 55.0 (51.2-58.8) | 0 (0-0) | 60.7 (57.5-63.9) | **<0.001** | **<0.001** | 0.026 | **<0.001** |
|  | n = 3128 |  |  |  |  |  |  |  |  |  |
|  | PG | 33.6 (32.0-35.2) | 0 (0-0) | 57.4 (54.0-60.8) | 0 (0-0) | 65.9 (62.9-68.9) | **<0.001** | **<0.001** | **<0.001** | **<0.001** |
|  | n = 3247 |  |  |  |  |  |  |  |  |  |
| **AHI/h** | PSG | 34.6 (33.7-35.4) | 37.4 (35.4-39.3) | 35.5 (33.6-37.4) | 34.4 (32.9-36.0) | 32.0 (30.5-33.5) | **<0.001** | **<0.001** | **0.004** | 0.188 |
|  | n = 3216 |  |  |  |  |  |  |  |  |  |
|  | PG | 28.4 (27.6-29.2) | 32.7 (30.8-34.6) | 31.3 (29.6-32.9) | 27.1 (25.7-28.6) | 24.0 (22.8-25.3) | **<0.001** | **<0.001** | **<0.001** | 0.258 |
|  | n = 3339 |  |  |  |  |  |  |  |  |  |
| **AHI > 30/h (%)** | PSG | 46.7 (45.0-48.4) | 49.9 (46.1-53.7) | 49.6 (45.9-53.3) | 47.5 (44.2-50.8) | 41.6 (38.5-44.7) | **0.002** | **<0.001** | **0.004** | 0.365 |
|  | n = 3216 |  |  |  |  |  |  |  |  |  |
|  | PG | 34.7 (33.1-36.3) | 42.8 (39.1-46.5) | 39.1 (35.8-42.4) | 34.0 (30.7-37.3) | 25.9 (23.2-28.6) | **<0.001** | **<0.001** | **<0.001** | 0.35 |
|  | n = 3339 |  |  |  |  |  |  |  |  |  |
| **ODI4 (events/h)** | PSG | 25.0 (24.2-25.9) | 26.6 (24.6-28.5) | 27.2 (25.3-29.2) | 23.7 (22.1-25.4) | 23.6 (22.0-25.1) | **0.004** | 0.018 | **0.004** | 0.645 |
|  | n = 3101 |  |  |  |  |  |  |  |  |  |
|  | PG | 24.0 (23.2-24.8) | 28.3 (26.3-30.2) | 27.2 (25.5-29.0) | 22.0 (20.5-23.5) | 19.7 (18.5-21.0) | **<0.001** | **<0.001** | **<0.001** | 0.446 |
|  | n = 2885 |  |  |  |  |  |  |  |  |  |
| **ODI4 > 30 (events/h; %)** | PSG | 31.2 (29.6-32.8) | 34.6 (31-38.2) | 34.5 (30.9-38.1) | 29.4 (26.3-32.5) | 28.2 (25.3-31.1) | 0.015 | 0.034 | 0.028 | 0.289 |
|  | n = 3101 |  |  |  |  |  |  |  |  |  |
|  | PG | 28.5 (26.9-30.1) | 35.5 (31.7-39.3) | 34.4 (31.0-37.8) | 25.8 (22.5-29.1) | 20.5 (17.8-23.2) | **<0.001** | **<0.001** | **<0.001** | 0.971 |
|  | n = 2885 |  |  |  |  |  |  |  |  |  |
| **Mean SaO_2_ (%)** | PSG | 93.2 (93.1-93.3) | 93.1 (92.8-93.3) | 93 (92.8-93.2) | 93.5 (93.3-93.6) | 93.1 (93.0-93.3) | **0.008** | 0.726 | 0.363 | 0.653 |
|  | n = 3216 |  |  |  |  |  |  |  |  |  |
|  | PG | 93.3 (93.2-93.4) | 93.1 (92.9-93.3) | 93.1 (92.9-93.2) | 93.5 (93.4-93.7) | 93.4 (93.3-93.5) | **<0.001** | 0.02 | **0.004** | 0.708 |
|  | n = 3339 |  |  |  |  |  |  |  |  |  |
| **Min SaO_2_ (%)** | PSG | 79.3 (78.9-79.6) | 78.4 (77.5-79.3) | 78.8 (78.0-79.6) | 79.7 (79-80.4) | 79.9 (79.2-80.5) | 0.019 | **0.007** | 0.036 | 0.523 |
|  | n = 3216 |  |  |  |  |  |  |  |  |  |
|  | PG | 79.4 (79.2-79.7) | 78.3 (77.6-79) | 78.3 (77.6-78.9) | 80.1 (79.5-80.6) | 80.7 (80.3-81.2) | **<0.001** | **<0.001** | **<0.001** | 0.987 |
|  | n = 3339 |  |  |  |  |  |  |  |  |  |
